# Genetic Algorithm Based on a New Similarity for Probabilistic Transformation of Belief Functions †

**DOI:** 10.3390/e24111680

**Published:** 2022-11-17

**Authors:** Yilin Dong, Lei Cao, Kezhu Zuo

**Affiliations:** 1 Department of Artificial Intelligence, Shanghai Maritime University, Shanghai 201306, China; 2 School of Cyber Science and Engineering, Southeast University, Nanjing 210096, China

**Keywords:** probabilistic transformation (PT), similarity measure, convergence analysis, belief functions (BFs)

## Abstract

Recent studies of alternative probabilistic transformation (PT) in Dempster–Shafer (DS) theory have mainly focused on investigating various schemes for assigning the mass of compound focal elements to each singleton in order to obtain a Bayesian belief function for decision-making problems. In the process of such a transformation, how to precisely evaluate the closeness between the original basic belief assignments (BBAs) and transformed BBAs is important. In this paper, a new aggregation measure is proposed by comprehensively considering the interval distance between BBAs and also the sequence inside the BBAs. Relying on this new measure, we propose a novel multi-objective evolutionary-based probabilistic transformation (MOEPT) thanks to global optimizing capabilities inspired by a genetic algorithm (GA). From the perspective of mathematical theory, convergence analysis of EPT is employed to prove the rationality of the GA used here. Finally, various scenarios in evidence reasoning are presented to evaluate the robustness of EPT.

## 1. Introduction

### 1.1. Background and Research Motivation

Since the pioneering work of Dempster and Shafer [1,2], known as Dempster–Shafer evidence theory (DST), belief functions are widely used in information fusion for decision making [3,4]. However, the computational complexity of reasoning with DST is one of the major points of criticism this formalism has to face. To overcome this difficulty, various approximating methods have been suggested that aim at reducing the number of focal elements in the frame of discernment (FoD) in order to maintain the tractability of computation. One common strategy is to simplify the FoD by removing or aggregating focal elements for approximating the original belief function [5]. Among these methods, probabilistic transformations (PTs) seem particularly desirable for reducing such computational complexity by means of assigning the mass of non-singleton elements to some singletons of the FoD [6,7]. The research on this probabilistic measure has received a lot of attention [8], and many efficient PTs have been proposed by scholars in recent years. Among them, a classical transformation, denoted as BetP [6], was usually adopted because it offered a compromise between the maximum of credibility (Bel) and the maximum of plausibility (Pl) for decision making. Unfortunately, BetP does not provide the highest probabilistic information content (PIC) [9], and Shenoy argued against BetP in his publication [10]. Sudano [11] also proposed series of alternatives and principles for them similar to BetP, which were called PrPl, PrBel and PrHyb. CuzzP [12], which was proposed by Cuzzolin in the framework of DST in 2009, showed its probabilistic transformation ability. Another novel transformation was proposed by Dezert and Smarandache in the framework of Dezert–Smarandache theory (DSmT) which was called Dezert–Smarandache probability (DSmP) [9], and comprehensive comparisons were made in [9] to prove the advantages of DSmP with respect to other PTs.

### 1.2. Challenges

Although various techniques have been proposed to evaluate PTs, these methods have limitations. On the one hand, the PIC or Shannon’s entropy criterion was applied to evaluate PTs, with less uncertainty (*clarity*) for the BBAs obtained from PTs being perferred in order to make decisions easily. However, Han et al. [13] and Bucci in [14] illustrated the irrationality of the overemphasizing of the Shannon entropy or PIC. On the other hand, from the perspective of *fidelity*, to characterize the difference between the transformed BBAs and original BBAs, various distances were applied. The most famous distance was $D_{J}$ [15], but it was not good enough to capture the difference between BBAs in some cases, and these can be seen in [16,17]. Thus, either the PIC or distance alone is not efficient and comprehensive enough to quantify all sorts of aspects of dissimilarity which inevitably need to be involved in PTs. To address incomprehensive evaluations, several two-dimensional measures [18] have been proposed in order to make sure that the results obtained by PTs are consistent in some manner with the original BBAs. Han in [19] proposed a 2D criteria which jointly uses distance and PIC measures to create a balance between fidelity and clarity. Liu [16] used a two-dimensional measure to effectively detect conflicts among the evidence. In [20], Liu proposed both a distance and a conflict coefficient based on probabilistic transformations BetP to characterize the dissimilarity, which are complementary in a certain sense. That aside, Deng [21] developed a new probability transformation method called the decision-based PT in belief functions theory. Following Deng’s idea [21], Zhao [22] proposed an importance-based PT to achieve the transformation of original basic belief assignments. Recently, Ma [23] integrated a fuzzy closeness and correlation coefficient to generate a new dissimilarity measure to characterize not only the difference between BBAs but also the divergence degree of the hypothesis that two BBAs support. By analyzing the mentioned existing methods, the relationship between the techniques of PTs and their corresponding evaluations are almost independent, except in [19]; that is to say, the methods of evaluation only assess the existing PTs instead of facilitating the development of novel PTs themselves.

### 1.3. Contributions

In this paper, we present a novel PT method based on a multi-objective algorithm (MOEPT) using reasonable and comprehensive two-dimensional criteria in order to capture the similarity in the process of a PT. This new method has some connections with the recent algorithm proposed in [19]. However, the main differences lie in the following aspects:

- The 2D criteria, PIC and Jousselme’s distance have pointed out its drawbacks in many references [17,24]. Thus, an efficient and different aggregation measure is proposed. Its novelty lies in considering the drawback of the past description of the distance between the evidence. In other words, up to now, most distances were defined according to the corresponding focal elements between two sources of evidence, and the sequence inside the assignments of the focal elements themselves was not considered. The sequence might also lead to dissimilarity, which is referred to as “self-conflict or self-contradiction” [25];
- More specific steps of evolutionary-based algorithms are given in detail. Aside from that, the convergence analysis of the MOEPT is illustrated to prove the rationality of using GAs. Moreover, some bugs are detected and fixed when using the MOEPT with traditional constraints;
- The specific application problem, target type tracking (TTT), is efficiently solved and discussed based on the proposed method with a novel simple constraint.

Compared with traditional PTs, global search replaces designing various assigning operators in classical PTs, and the evaluation criteria are embedded into an MOEPT to provide important guidance for the searching procedure. Specifically, masses of singletons are randomly generated in an evolutionary-based framework, which needs to satisfy with the basic constraints for probability distributions in evidence reasoning. Additionally, an assessment factor is presented to assess the best individual in all populations by a special objective function (desirable evaluation criteria). The simulation results in 4D FoD test cases show that in these problems, the proposed MOEPT was able to outperform other PTs from the perspective of the 2D criteria. Moreover, we propose a simple constraint-handling strategy within the MOEPT that is well-suited for two-target type tracking (2-TTT) problems, which to some extent encourages the application of MOEPTs to more complex and real-world decision-making problems.

The remainder of this paper is structured as follows. In Section 2, we briefly summarize the basis of DST. The new aggregation measure is proposed in Section 3. In Section 4, multi-objective evolutionary algorithms (EAs) based on a two-dimensional objective function are proposed. In Section 5, several examples and comprehensive comparisons are carried out. A simple pattern recognition problem and also a target type tracking problem are presented and solved in detail at the end of this section. The conclusions are drawn in Section 6.

## 2. Basis of Belief Functions

In this section, we introduce the belief function terminology of DST and the notations used in the sequel to this paper.

### 2.1. DST Basis

In DST [2], the elements $\theta_{i}$ (i=1,…,N) of the frame of discernment (FoD) $\Theta ≜\{\theta_{1},\ldots ,\theta_{N}\}$ must be mutually exhaustive and exclusive. The power set of the FoD is denoted as $2^{\Theta }$, and a basic belief assignment (BBA), also called a mass function, is defined by the mapping: $2^{\Theta }\to \left[0,1\right]$, which satisfies m(∅)=0 and

(1) $$\sum_{A\subseteq 2^{\Theta }}m\left(A\right)=1$$

where m(A) is defined as the BBA of *A*. The element *A* is called a focal element of m(.) if m(A)>0. The belief and plausibility functions, which are in one-to-one mapping with the BBA m(.), are defined for all A⊆Θ by

(2) $$Bel\left(A\right)=\sum_{B\in 2^{\Theta }|B\subseteq A}m\left(B\right)$$

(3) $$Pl\left(A\right)=1-Bel\left(\bar{A}\right)=\sum_{A,B\in 2^{\Theta }|A\cap B\neq \emptyset }m\left(B\right)$$

where $\bar{A}≜\Theta \backslash A$ is the complement to *A* in Θ. The belief interval [Bel(A),Pl(A)] represents the uncertainty committed to *A*, and the bounds of this interval are usually interpreted as lower and upper bounds of the unknown (possibly subjective) probability of *A*.

In order to fuse *n* bodies Of evidence (BOEs), Dempster’s rule of combination is usually used in the DST framework. The combination of n distinct BOEs is achieved as follows:

(4) $$m\left(A\right)=\left\{\begin{array}{cc} 0, & \;\;ifA=\emptyset \\ \frac{\sum_{\cap A_{i}=A}\prod_{1\leq i\leq n}m_{i}\left(A_{i}\right)}{\sum_{\cap A_{i}\neq \emptyset }\prod_{1\leq i\leq n}m_{i}\left(A_{i}\right)}, & \;\;ifA\neq \emptyset \end{array}\right.$$

### 2.2. Classical Probabilistic Transformations

The efficiency of a probabilistic transformation (PT) in the field of decision making was analyzed in depth by Smets [6]. Various PTs have been proposed in the open literature such as BetP [6,26], CuzzP [12], DSmP [9], PrBP1 and PrBP2 [27], as well as Cobb and Shenoy’s normalization of plausibility [10]. The simple and classical transformation (BetP) is briefly recalled in this subsection.

#### Pignistic Transformation (BetP)

Smets in [6,26] first proposed pignistic (also called *betting*) probability to make decisions, which aims to transfer the mass of belief of each non-specific element onto the singletons. The classical pignistic probability is defined as BetP(∅)=0, and $\forall A\in 2^{\Theta }\backslash \left\{\emptyset \right\}$:

(5) $$BetP\left(\theta_{i}\right)≜\sum_{A\subseteq 2^{\Theta },A\neq \emptyset }\frac{\left|\theta_{i}\cap A\right|}{\left|A\right|}\frac{m(A)}{1-m(\emptyset )}$$

Because in Shafer’s framework m(∅)=0, Equation (5) can simply be rewritten for any singleton $\theta_{i}\in \Theta$ as

(6) $$\begin{array}{c} BetP\left(\theta_{i}\right)=\sum_{B\in 2^{\Theta },\theta_{i}\subseteq B}\frac{1}{\left|B\right|}m\left(B\right)=m\left(\theta_{i}\right)+\sum_{B\in 2^{\Theta },\theta_{i}\subset B}\frac{1}{\left|B\right|}m\left(B\right) \end{array}$$

### 2.3. Distance Proposed by Han and Dezert $d_{BI}^{E}$

The Jousselme’s distance, which was widely denoted as $D_{J}$ in [15], was applied in many recent works [19,28], but the particular choice of the $D_{J}$ distance is not a very good choice because one knows that the $D_{J}$ distance has bad behavior. This was clearly explained recently in [17,24]. Assuming that two independent BBAs $m_{1}\left(\cdot \right)$ and $m_{2}\left(\cdot \right)$ are defined on $\Theta =\{\theta_{1},\theta_{2},\ldots ,\theta_{n}\}$, for each focal element $\theta_{i}\in \Theta \;\left(i=1,2,\ldots ,2^{n}-1\right)$, belief intervals of $\theta_{i}$ for $m_{1}\left(\cdot \right)$ and $m_{2}\left(\cdot \right)$ can be calculated, which are denoted by $\left[Bel_{1}\left(\theta_{i}\right),Pl_{1}\left(\theta_{i}\right)\right]$ and $\left[Bel_{2}\left(\theta_{i}\right),Pl_{2}\left(\theta_{i}\right)\right]$, respectively. The strict distance between the interval numbers [a,b] and [c,d] is defined by [29]

(7) $$d^{I}\left(\left[a,b\right],\left[c,d\right]\right)=\sqrt{{\left[\frac{a+b}{2}-\frac{c+d}{2}\right]}^{2}+\frac{1}{3}{\left[\frac{b-a}{2}-\frac{d-c}{2}\right]}^{2}}$$

Therefore, we can calculate the distance between $BI_{1}\left(\theta_{i}\right):\left[Bel_{1}\left(\theta_{i}\right),Pl_{1}\left(\theta_{i}\right)\right]$ and $BI_{2}\left(\theta_{i}\right):\left[Bel_{2}\left(\theta_{i}\right),Pl_{2}\left(\theta_{i}\right)\right]$ according to Equation (7). Thus, we can obtain a total of $2^{N}-1$ belief interval distance values for all $\theta_{i}\in \Theta$. Aside from that, the Euclidean family belief interval-based distance $d_{BI}^{E}$ can be rewritten as

(8) $$d_{BI}^{E}\left(m_{1},m_{2}\right)=\sqrt{N_{c}\cdot \sum_{i=1}^{2^{n}-1}{\left[d^{I}\left(BI_{1}\left(\theta_{i}\right),BI_{2}\left(\theta_{i}\right)\right)\right]}^{2}}$$

Here, $N_{c}=1/2^{n}-1$ is the normalization factor. In this paper, we regard $d_{BI}^{E}$ as one criterion for evaluating the degree of similarity (similarity representing the degree of difference between the original BBAs and the transformed ones in [30]) between the original BBAs and the transformed ones.

## 3. New Evidence for Similarity Characterization

As mentioned in previous section, those distances (i.e., Jousselme’s distance [28]) and other metrics such as the PIC [31] or entropy [27] were widely applied to measure the degree of “similarity or dissimilarity” between BBAs. However, only the corresponding focal elements (or the relevant focal element set) between two sources of evidence are described or characterized. This one-sided view does not consider the order of size of the assignment of each focal element in the evidence, which might lead to “self-conflict or self-contradiction”. To consider such “information” produced by the evidence itself, here, a new evidence similarity measure is defined between two evidential sources according to the order of size of the assignment. Prior to this, to give this new similarity measure, first we define the order correlation coefficient between two sets of data:

**Definition** **1.** *[32] Given two sets of data $\left\{x_{1},x_{2},\ldots ,x_{n}\right\}$ and $\left\{y_{1},y_{2},\ldots ,y_{n}\right\}$, here, $x_{1},x_{2},\ldots ,x_{n}$ and $y_{1},y_{2},\ldots ,y_{n}$ are in an ascending order. After sorting, two sets of data $x_{p_{1}},x_{p_{2}},\ldots ,x_{p_{n}}$ and $y_{q_{1}},y_{q_{2}},\ldots ,y_{q_{n}}$ meet $x_{p_{1}}\leq x_{p_{2}}\leq \ldots \leq x_{p_{n}}$ and $y_{q_{1}}\leq y_{q_{2}}\leq \ldots \leq y_{q_{n}}$, respectively, for each $p_{i}$ and index their positions from $q_{1},q_{2},\ldots ,q_{n}$, assuming it is $q_{j}$; that is, $q_{j}=p_{i}$. Note that j=f(i), and the correlation coefficient is*

(9) $$\mu =\frac{\sum_{i=1}^{n}{\left(i-j\right)}^{2}}{\sum_{i=1}^{n}{\left[n-\left(i-1\right)-i\right]}^{2}}$$

*This satisfies 0≤μ≤1. When μ=0, the convergence of two sets of data is the largest. When μ=1, this is reversed.*

### 3.1. The Consistency of Focal Elements between Two BOEs

**Definition** **2.** *[32] For any two sources of evidence (i.e., $S_{1}$ and $S_{2})$, $m_{1}\left(\cdot \right)$ and $m_{2}\left(\cdot \right)$ are the basic belief assignments over the discernment framework *Θ* of a size n. The number of focal elements and the focal elements of $m_{1}\left(.\right)$ and $m_{2}\left(.\right)$ can be different. We denote $X_{i}$ and $Y_{i}$ as the indexes of the focal elements whose masses are sorted by increasing order. The similarity function of the evidence to characterize the order of the size of the assignments over the subsets is as follows:*

(10) $$Sim_{seq}\left(m_{1},m_{2}\right)=1-\frac{\sum_{i=1}^{n}{\left(X_{i}-Y_{i}\right)}^{2}}{\sum_{i=1}^{n}{\left[n+1-2i\right]}^{2}}$$

As we all know, if there is a similarity function $Sim(m_{i},m_{j})$, which is the characterization of distance between any two evidence sources, then the following four basic conditions must be satisfied:

- Symmetry: $\forall m_{i}\left(\cdot \right),m_{j}\left(\cdot \right),Sim\left(m_{i},m_{j}\right)=Sim\left(m_{j},m_{i}\right)$;
- Consistency: ∀m(·),Sim(m,m)=1;
- Nonnegative: $\forall m_{i}\left(\cdot \right),m_{j}\left(\cdot \right),0\leq Sim\left(m_{i},m_{j}\right)\leq 1$;
- Triangle inequality: Sim(X,Y)+Sim(Y,Z)≥Sim(X,Z).

According to previous work [32], it is easy to find that $Sim_{seq}$ satisfies symmetry, consistency and nonnegativity, but the last important condition is lost. Therefore, we prove the triangle inequality property of $Sim_{seq}$ here:

**Proof.**  Based on Equation (10), the triangle inequality can be rewritten as follows: $1-\frac{\sum_{i=1}^{n}{\left(X_{i}-Z_{i}\right)}^{2}}{\sum_{i=1}^{n}{\left[n+1-2i\right]}^{2}}\leq 1-\frac{\sum_{i=1}^{n}{\left(X_{i}-Y_{i}\right)}^{2}}{\sum_{i=1}^{n}{\left[n+1-2i\right]}^{2}}+1-\frac{\sum_{i=1}^{n}{\left(Y_{i}-Z_{i}\right)}^{2}}{\sum_{i=1}^{n}{\left[n+1-2i\right]}^{2}}$⇒ $\sum_{i=1}^{n}{\left[n+1-2i\right]}^{2}\geq \sum_{i=1}^{n}{\left(X_{i}-Y_{i}\right)}^{2}+\sum_{i=1}^{n}{\left(Y_{i}-Z_{i}\right)}^{2}-\sum_{i=1}^{n}{\left(X_{i}-Z_{i}\right)}^{2}$.

Here, ***X***, ***Y*** and ***Z*** denote vectors ⇒$\sum_{i=1}^{n}{\left[n+1-2i\right]}^{2}\geq {\left(X-Y\right)}^{T}\left(X-Y\right)+{\left(Y-Z\right)}^{T}\left(Y-Z\right)-{\left(X-Z\right)}^{T}\left(X-Z\right)$.

According to the squared sum formula (SSF) (squared sum formula of a natural number: $1^{2}+2^{2}+3^{2}+\ldots +n^{2}=\frac{n(n+1)(2n+1)}{6}$; squared sum formula of an odd number: $1^{2}+3^{2}+5^{2}+\ldots +{\left(2n-1\right)}^{2}=\frac{1}{3}n\left(4n^{2}-1\right)$; squared sum formula of an even number: $2^{2}+4^{2}+\ldots +{\left(2n\right)}^{2}=\frac{2}{3}n\left(n+1\right)\left(2n+1\right)$), we have

$$\begin{array}{cc} \; & \sum_{i=1}^{n}{\left[n+1-2i\right]}^{2}=\sum_{i=1}^{n}\left[{\left(n+1\right)}^{2}+4i^{2}-4\left(n+1\right)i\right]=\\ \; & =n{\left(n+1\right)}^{2}+4*\frac{1}{6}n\left(n+1\right)\left(2n+1\right)-4\left(n+1\right)\frac{n(n+1)}{2}\\ \; & =\frac{2}{3}n\left(n+1\right)\left(2n+1\right)-n{\left(n+1\right)}^{2}\\ \; & =n\left(n+1\right)\cdot \left[\frac{4}{3}n+\frac{2}{3}-n-1\right]\\ \; & =\frac{1}{3}\cdot n\cdot \left(n^{2}-1\right) \end{array}$$

Because $1\leq X_{i}\leq n,1\leq Y_{i}\leq n,1\leq Z_{i}\leq n$, we obtain the following:

${\left(X-Y\right)}^{T}\left(X-Y\right)+{\left(Y-Z\right)}^{T}\left(Y-Z\right)-{\left(X-Z\right)}^{T}\left(X-Z\right)\leq 1+{\left(k-1\right)}^{2}+{\left(k-3\right)}^{2}+\ldots$

- When $n-1=2k\Rightarrow k=\frac{n-1}{2}$, $1+{\left(k-1\right)}^{2}+{\left(k-3\right)}^{2}+\ldots =\frac{1}{6}\cdot k\left(k+1\right)\left(k+2\right)$, and thus
  $$\begin{array}{cc} \; & \sum_{i=1}^{n}{\left[n+1-2i\right]}^{2}-\left[{\left(X-Y\right)}^{T}\left(X-Y\right)+{\left(Y-Z\right)}^{T}\left(Y-Z\right)-{\left(X-Z\right)}^{T}\left(X-Z\right)\right]\\ \; & \geq \frac{1}{3}\cdot n\cdot \left(n^{2}-1\right)-\frac{1}{6}\cdot k\left(k+1\right)\left(k+2\right)\\ \; & =\frac{1}{3}\cdot n\cdot \left(n^{2}-1\right)-\frac{1}{6}\cdot \frac{n-1}{2}\left(\frac{n-1}{2}+1\right)\left(\frac{n-1}{2}+2\right)\\ \; & =\frac{1}{3}\cdot n\cdot \left(n^{2}-1\right)-\frac{1}{6}\cdot \frac{n-1}{2}\cdot \frac{n+1}{2}\cdot \frac{n+3}{2}\\ \; & =\left(n^{2}-1\right)\cdot \left(\frac{1}{4}n+\frac{1}{6}\right) \end{array}$$
  Because n≥2, thus
  $\sum_{i=1}^{n}{\left[n+1-2i\right]}^{2}-\left[{\left(X-Y\right)}^{T}\left(X-Y\right)+{\left(Y-Z\right)}^{T}\left(Y-Z\right)-{\left(X-Z\right)}^{T}\left(X-Z\right)\right]\geq 0$⇒${\mathrm{Sim}}_{\mathrm{seq}}$ satisfy triangle inequality;
- When $n-1=2k-1\Rightarrow k=\frac{n}{2}$, $1+{\left(k-1\right)}^{2}+{\left(k-3\right)}^{2}+\ldots =\frac{1}{3}\cdot k\cdot \left(4k^{2}-1\right)$, thus
  $$\begin{array}{cc} \; & \sum_{i=1}^{n}{\left[n+1-2i\right]}^{2}-\left[{\left(X-Y\right)}^{T}\left(X-Y\right)+{\left(Y-Z\right)}^{T}\left(Y-Z\right)-{\left(X-Z\right)}^{T}\left(X-Z\right)\right]\\ \; & \geq \frac{1}{3}\cdot n\cdot \left(n^{2}-1\right)-\frac{1}{3}\cdot k\cdot \left(4k^{2}-1\right)\\ \; & =\frac{1}{3}\cdot n\cdot \left(n^{2}-1\right)-\frac{1}{3}\cdot \frac{n}{2}\cdot \left(4{\left(\frac{n}{2}\right)}^{2}-1\right)\\ \; & =\frac{1}{3}\cdot n\cdot \left(n^{2}-1\right)-\frac{n}{6}\cdot \left(n^{2}-1\right)\\ \; & =\frac{n}{6}\cdot \left(n^{2}-1\right) \end{array}$$
  Because n≥2, thus $\sum_{i=1}^{n}{\left[n+1-2i\right]}^{2}-\left[{\left(X-Y\right)}^{T}\left(X-Y\right)+{\left(Y-Z\right)}^{T}\left(Y-Z\right)-{\left(X-Z\right)}^{T}\left(X-Z\right)\right]\geq 0$⇒${\mathrm{Sim}}_{\mathrm{seq}}$ satisfies the triangle inequality.

   □

**Definition** **3.** *[32] For any two sources of evidence (i.e., $S_{1}$ and $S_{2}$), $m_{1}\left(\cdot \right)$ and $m_{2}\left(\cdot \right)$ are the basic belief assignments over n focal elements in the discernment framework *Θ* (note that the BBA of each subproposition might be same). Assume that the $s_{1}$ subpropositions’ BBAs are the same in $m_{1}\left(\cdot \right)$, and the $s_{2}$ subpropositions’ BBAs are the same in $m_{2}\left(\cdot \right)$ Herein, $X_{i}$ and $Y_{i}$ are the serial numbers according to the order of the size of the subpropositions’ BBAs, where the subscript i indicates the ith subproposition, due to the BBAs of some subpropositions being the same. For the evidence $S_{1}$, there might be $s_{1}$ kinds of sorts. For $S_{2}$, there might be $s_{2}$ kinds of sorts. Therefore, there are $s_{1}\times s_{2}$ kinds of sorts for $S_{1}$ and $S_{2}$. The similarity measure functions are redefined in this case as follows:*

(11) $$Sim_{seq}^{\prime }\left(m_{1},m_{2}\right)=1-\frac{\sum_{t=1}^{s_{1}s_{2}}\sum_{i=1}^{n}{\left(X_{i}^{t}-Y_{i}^{t}\right)}^{2}}{s_{1}s_{2}\left(\sum_{i=1}^{n}{\left(n+1-2i\right)}^{2}\right)}$$

*Similarly, it is easy to prove that $Sim_{seq}^{\prime }\left(m_{X},m_{Y}\right)$ is a similarity measure function.*

**Example** **1.** ***Bayesian*** ***BBAs:*** *Assuming two kinds of evidence $m_{1}=\left\{\theta_{1},\theta_{2},\theta_{3}\right\}=\left\{0,0.1,0.9\right\}$ and $m_{2}=\left\{\theta_{1},\theta_{2},\theta_{3}\right\}=\left\{0.9,0.1,0\right\}$, then $m_{1}$ and $m_{2}$ are sorted from small to large so that $X\left(m_{1}\right)=\left\{\theta_{1},\theta_{2},\theta_{3}\right\}=\left[1,2,3\right]$ and $Y\left(m_{2}\right)=\left\{\theta_{3},\theta_{2},\theta_{1}\right\}=\left[3,2,1\right]$, respectively. Thus, we can calculate the similarity measure based on Equation (10):*

$$\begin{array}{cc} \; & Sim_{seq}\left(m_{1},m_{2}\right)=\\ \; & 1-\frac{{\left(1-3\right)}^{2}+{\left(2-2\right)}^{2}+{\left(3-1\right)}^{2}}{{\left(3+1-2*1\right)}^{2}+{\left(3+1-2*2\right)}^{2}+{\left(3+1-2*3\right)}^{2}}=0 \end{array}$$

*According to $Sim_{seq}\left(m_{1},m_{2}\right)$, we can find that $m_{1}$ and $m_{2}$ are completely different and lacking similarity.*

### 3.2. The Inconsistency of the Focal Elements between Two BOEs

How do we calculate $Sim_{seq}$ when the focal elements in BBAs are different? Let us consider the following example and put forward a different way compared with that in [32]:

**Example** **2.** ***General BBAs:*** *Assuming two types of evidence $m_{1}=\left\{\theta_{1},\theta_{2}\cup \theta_{3},\theta_{2}\cup \theta_{4},\theta_{1}\cup \theta_{3}\cup \theta_{4}\right\}=\left\{0.3,0.2,0.2,0.3\right\}$ and $m_{2}=\left\{\theta_{1},\theta_{1}\cup \theta_{3},\theta_{3},\theta_{2}\cup \theta_{3}\cup \theta_{4},\theta_{1}\cup \theta_{2}\cup \theta_{3}\cup \theta_{4}\right\}=${0.4,0.1,0.1,0.2,0.2}.*

*Borrowing ideas from Dezert ($d_{BI}^{E}$) in [24], for each focal element $\theta_{i}\in \Theta \left(i=1,2,\ldots ,2^{n}-1\right)$, the belief intervals of $\theta_{i}$ for $m_{1}\left(\cdot \right)$ and $m_{2}\left(\cdot \right)$ can be calculated, which are denoted by $\left[Bel_{1}\left(\theta_{i}\right),Pl_{1}\left(\theta_{i}\right)\right]$ and $\left[Bel_{2}\left(\theta_{i}\right),Pl_{2}\left(\theta_{i}\right)\right]$, respectively. According to the theory of evidence, the width of such an interval as $\left[Bel_{1}\left(\theta_{i}\right),Pl_{1}\left(\theta_{i}\right)\right]$ represents the degree of uncertainty for the corresponding focal element $\theta_{i}$. Therefore, $X_{i}$ and $Y_{i}$ in Equation (10) are obtained, which refer to the index of the width of the interval for each focal element, whose values are sorted in increasing order. The steps of this mechanism are illustrated as follows:*

- *Step 1:*
*$\left[Bel_{1}\left(\theta_{1}\right),Pl_{1}\left(\theta_{1}\right)\right]=\left[0.3,0.6\right]$, $\left[Bel_{1}\left(\theta_{2}\right),Pl_{1}\left(\theta_{2}\right)\right]=\left[0,0.4\right]$,*
*$\left[Bel_{1}\left(\theta_{3}\right),Pl_{1}\left(\theta_{3}\right)\right]=\left[0,0.5\right]$, $\left[Bel_{1}\left(\theta_{4}\right),Pl_{1}\left(\theta_{4}\right)\right]=\left[0,0.5\right]$,*
*$\left[Bel_{2}\left(\theta_{1}\right),Pl_{2}\left(\theta_{1}\right)\right]=\left[0.4,0.7\right]$, $\left[Bel_{2}\left(\theta_{2}\right),Pl_{2}\left(\theta_{2}\right)\right]=\left[0,0.4\right]$,*
*$\left[Bel_{2}\left(\theta_{3}\right),Pl_{2}\left(\theta_{3}\right)\right]=\left[0.1,0.6\right]$, $\left[Bel_{2}\left(\theta_{4}\right),Pl_{2}\left(\theta_{4}\right)\right]=\left[0,0.4\right]$;*
- *Step 2: The parameter ς denotes the width of the belief interval, where $\varsigma_{1}\left(\theta_{1}\right)=Pl_{1}\left(\theta_{1}\right)-Bel_{1}\left(\theta_{1}\right)=0.3$, $\varsigma_{1}\left(\theta_{2}\right)=0.4$, $\varsigma_{1}\left(\theta_{3}\right)=0.5$, $\varsigma_{1}\left(\theta_{4}\right)=0.5$, $\varsigma_{2}\left(\theta_{1}\right)=0.3$, $\varsigma_{2}\left(\theta_{2}\right)=0.4$, $\varsigma_{2}\left(\theta_{3}\right)=0.5$ and $\varsigma_{2}\left(\theta_{4}\right)=0.4$;*
- *Step 3: $X_{1}$ and $Y_{1}$ are the indexes of focal elements whose ς values are sorted in increasing order, where $X_{1}=\left\{1,2,3,3\right\}$ and $Y_{1}=\left\{1,2,3,2\right\}$;*
- *Step 4: $Sim_{seq}$ is calculated based on Equation (10).*

To consider the influence of the distance of the evidence, here, based on $d_{BI}^{E}$ in Equation (8), we propose a new similarity measure which is presented as follows:

(12) $$Cim\left(m_{i},m_{j}\right)=w_{1}\cdot d_{BI}^{E}\left(m_{i},m_{j}\right)+w_{2}\cdot ϝ\left(Sim_{seq}\left(m_{i},m_{j}\right)\right)$$

where $w_{1}=w_{2}=0.5.$ and ϝ(·) is the decreasing function within the interval [0,1], for which in this paper $ϝ\left(\cdot \right)=1-x^{2}$. That aside, it is easy to prove that the improved measure function $Cim(m_{i},m_{j})$ is still a similarity measure function. This is because the final metric between two similarity measure functions still meets the definition of a similarity measure function.

Additionally, to consider the normalization of Equation (12), it can be rewritten as follows:

(13) $$C_{norm}\left(m_{i},m_{j}\right)=w_{1}*\left(d_{BI}^{E*}\left(m_{i},m_{j}\right)\right)+w_{2}*\left(ϝ\left(Sim_{seq}^{*}\left(m_{i},m_{j}\right)\right)\right)$$

where $d_{BI}^{E*}\left(\hat{m}\right)=\frac{d_{BI}^{E}\left(m_{i},m_{j}\right)-min\left({\mathrm{vector}}_{1}\right)}{max\left({\mathrm{vector}}_{1}\right)-min\left({\mathrm{vector}}_{1}\right)}$, $Sim_{seq}^{*}\left(m_{i},m_{j}\right)=\frac{Sim_{seq}\left(m_{i},m_{j}\right)-min\left({\mathrm{vector}}_{2}\right)}{max\left({\mathrm{vector}}_{2}\right)-min\left({\mathrm{vector}}_{2}\right)}$,

${\mathrm{vector}}_{1}=\left(D_{1}\left(m_{i},m_{1}\right),D_{2}\left(m_{i},m_{2}\right),\ldots ,D_{j}\left(m_{i},m_{j}\right)\right)$ and

${\mathrm{vector}}_{2}=\left(Sim_{1}\left(m_{i},m_{1}\right),Sim_{2}\left(m_{i},m_{2}\right),\ldots ,Sim_{j}\left(m_{i},m_{j}\right)\right)$.

## 4. Multi-Objective Evolutionary Algorithm Based on Two-Dimensional Criteria

In this section, we regard a PT as a general multi-objective problem consisting of two objectives also involved in a number of inequality and equality constraints. Then, a corresponding optimization model is proposed for selecting the *best* Bayesian BBA in the set of candidates.

### 4.1. Multiple-Objective Evolutionary-Based Probabilistic Transformation

The idea to approximate any BBA into a Bayesian BBA (i.e., a subjective probability measure) using the minimization of the Shannon entropy under compatibility constraints was proposed recently by Han et al. in [13,19] using *on-the-shelf* optimization techniques. In this paper, we present in detail a new optimization method to achieve this PT based on a random evolutionary algorithm to acquire minimization of the new aggregation criteria, and this new comprehensive criteria represents different aspects of information in BBAs. For example, the *conflict coefficient* represents the degree of similarity in *conflict* between transformed BBAs and original BBAs (in other words, the more conflicts that exist between two BBAs, the less similarity they have). In addition, $d_{BI}^{E}$ represents the interval distance between the original BBAs and the transformed ones.

Let us assume that the FoD of the original BBA m(.) approximated by a Bayesian BBA is $\Theta ≜\{\theta_{1},\theta_{2},\ldots ,\theta_{N}\}$. The MOEPT method consists of the following steps, which are derived from GAs:

- Step 0 (setting parameters): Assume $t_{\max}$ is the maximum number of iterations, $n_{\max}$ is the population size in each iteration, $P_{s}$ is the selection probability, $P_{c}$ is the crossover probability, and $P_{m}$ is the mutation probability.
- Step 1 (population generation and encoding mechanism): A set $P_{t}$ of $j=1,2,\ldots ,n_{\max}$ random probability values $P_{t}^{j}=\left\{P^{j}\left(\theta_{1}\right),\ldots ,P^{j}\left(\theta_{N}\right)\right\}$ is generated such that the constraints in Equations (14)–(16) for $j=1,2,\ldots ,n_{\max}$ are satisfied in order to make each random set of probabilities $P_{t}^{j}$ compatible with the original or target BBA m(.) to approximate. (The lower (Bel) and upper (Pl) limits of each focal element are calculated using Equations (2) and (3) based on the value of m(·).) In other words, we have
  (14) $$\begin{array}{cc} \; & P^{j}\left(\theta_{i}\right)\in \left[0,1\right],\;i=1,2,\ldots ,N\;\;\;\;\;\;\;\;\;\;\;\;\;\;\;\;\;\;\;\;\;\; \end{array}$$
  (15) $$\begin{array}{cc} \; & \sum_{i=1}^{N}P^{j}\left(\theta_{i}\right)=1\;\;\;\;\;\;\;\;\;\;\;\;\;\;\;\;\;\;\;\;\;\;\;\;\;\;\;\;\;\;\;\;\;\;\;\;\;\;\;\;\;\;\;\;\;\;\;\;\;\;\;\;\; \end{array}$$
  (16) $$\begin{array}{cc} \; & Bel\left(\theta_{i}\right)\leq P^{j}\left(\theta_{i}\right)\leq Pl\left(\theta_{i}\right),\;i=1,2,\ldots ,N \end{array}$$
- Step 2 (fitness assignment): For each probability set $P_{t}^{j}$, ($j=1,2,\ldots ,n_{\max}$), we compute its fitness value *F* based on Equation (13). More precisely, one takes $F\left(P_{t}^{j}\right)=C_{norm}\left(m\left(\cdot \right),P_{t}^{j}\right)$.
- Step 3 (best approximation of m(.)): The best probability set $P_{t}^{j_{\mathrm{best}}}$ with a minimum fitness value is sought, and its associated index $j_{\mathrm{best}}$ is stored in BestIndividual and IndexofBestIndividual.
- Step 4 (selection, crossover and mutation): The tournament selection, crossover and mutation operators drawn from the evolutionary theory framework [33] are implemented to create the associated offspring population $P_{t}^{\prime }$ based on the parent population $P_{t}$. If $F\left(P_{t}^{j_{\mathrm{best}}}\right)\leq F\left(P_{t}^{\prime j_{\mathrm{best}}}\right)$, then the BestIndividual remains unchanged; otherwise, $\mathrm{Best}\;\mathrm{Individual}=P_{t}^{\prime j_{\mathrm{best}}}$.
  - Crossover operator: The crossover operator is one of the most important operators in the genetic algorithm. The crossover operation is conducted for the selected pairs of individuals. The feasibility condition of each individual is described as follows. The value of each subsegment must be between 0 and 1, and the sum of the individuals should be 1. Although the initial population is formed in a way that all individuals are feasible and correct, using the standard crossover operators leads to defective sub-segments, and a normalization procedure is needed for such a situation. Consider the following two individuals to be parents: X=(0.1,0.2,0.3,|0.4) and Y=(0.2,0.2,0.1,|0.5). (Here, the vertical bar represents the intersection point with the crossover operator.) With the single-point classic crossover operator, the following offspring will be produced: $X^{\prime }=\left(0.1,0.2,0.3,0.5\right)$ and $Y^{\prime }=\left(0.2,0.2,0.1,0.4\right)$, where $\sum_{j=1}^{4}X_{j}^{\prime }$ is equal to 1.1, which is greater than 1, and $\sum_{j=1}^{4}Y_{j}^{\prime }$ is equal to 0.9, which is less than 1. Therefore, $X^{\prime }$ and $Y^{\prime }$ have defective values for which a normalization factor is needed, which leads to the following:
    $X^{\prime \prime }=\frac{X^{\prime }}{\sum_{j=1}^{4}X_{j}^{\prime }}=\left(0.1/1.1,0.2/1.1,0.3/1.1,0.5/1.1\right)$,
    $Y^{\prime \prime }=\frac{Y^{\prime }}{\sum_{j=1}^{4}Y_{j}^{\prime }}=\left(0.2/0.9,0.2/0.9,0.1/0.9,0.4/0.9\right)$.
  - Mutation operator: The mutation operator randomly alters the value of a sub-segment. After applying the mutation operator, normalization of the changed individuals is required. The normalization will be performed in a similar way to the crossover operator.
- Step 5 (stopping MOEPT): Steps 1–4 illustrate the *t*th iteration of the MOEPT method. If $t\geq t_{\max}$, then the MOEPT method is complete; otherwise, another iteration must be performed by taking t+1=t and going back to step 1.

**Figure 1:**
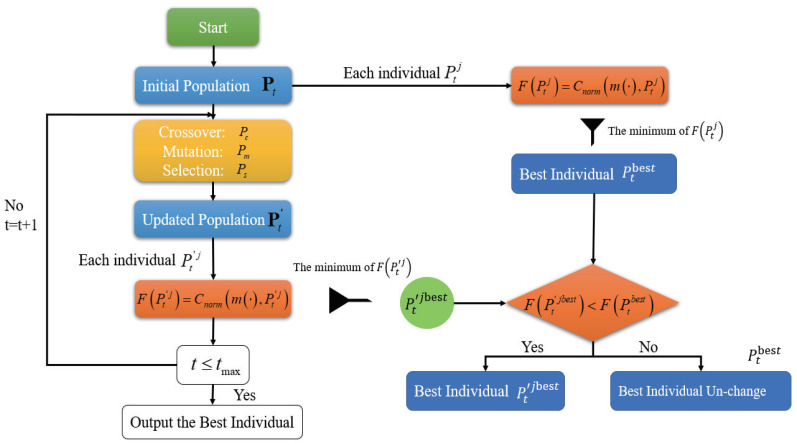
Scheme of MOEPT algorithm.

The scheme of the MOEPT method is shown in Figure 1, and its pseudo-code is given in Algorithm 1.
**Algorithm 1:** Multi-Objective Evolutionary-Based PT (MOEPT).1:Define Stopping Criteria, ($t\leq t_{\max}$); population Size $n_{\max}$ for each iteration; crossover probability $P_{c}$, mutation probability $P_{m}$ and selection probability $P_{s}$.  2:Generate an initial random population $P_{t}$ of consistent probabilities $P_{t}^{j}$ with m(.).  3:For each individual $P_{t}^{j}$ in $P_{t}$ do  4:  Calculate Fitness $F\left(P_{t}^{j}\right)=C_{norm}\left(m\left(\cdot \right),P_{t}^{j}\right)$ of $P_{t}^{j}$  5:  Store the best individual $P_{t}^{j_{\mathrm{best}}}$  6:End  7:Repeat:  8:  Crossover: exchange parts of individuals with probability $P_{c}$  9:  Mutation: mutate the child individuals with probability $P_{m}$  10:  Selection: Select individuals based on fitness according to $P_{s}$  11:  After these three sub-steps, the updated population $P_{t}^{\prime }$ is obtained  12:  Calculate the fitness of individuals of $P_{t}^{\prime }$, and store the best individual ${P_{t}^{\prime }}^{j_{best}}$  13:  If $F\left(P_{t}^{j_{\mathrm{best}}}\right)\leq F\left({P_{t}^{\prime }}^{j_{best}}\right)$  14:    Best-Individual remains unchanged  15:  else  16:    Best-Individual $=P_{t}^{\prime j_{\mathrm{best}}}$  17:If $t\geq t_{\max}$ then stops, otherwise t+1→t and go back to line 7  

### 4.2. Convergence Analysis

In order to mathematically prove the feasibility of an MOEPT, convergence analysis of our algorithm is given. First, we give a simplified description of the algorithm and also its symbolic representation for simplicity:

- Encoding mechanism: The size of the population is $n_{max}$, the length of individual (chromosome) is N, and the initial population is $P_{1}$;
- Retain the best individual directly for the next generation;
- Randomly select the other non-optimal individuals in $P_{t}$ to cross over so as to form the intermediate population $Y_{t}$;
- The population $Y_{t}$ is mutated to form a population $V_{t}$;
- The better individuals in the population $V_{t}$ are selected as the new generation population $P_{t}$.

Specifically, three operators (crossover operator, mutation operator and selection operator) can be described by the transition probability as follows:

- Crossover operator: For a single-point crossover, a new individual *k* is produced based on their parents: individuals *i* and *j*:
  (17) $$P_{C}^{t}\left(i\times j,k\right)=\left\{\begin{array}{c} \left|k\right|p_{c}/N,k\neq i,j\\ \left(1-p_{c}\right)+\left|k\right|p_{c}/N,k=i \end{array}\right.$$
  where |k| is the number of individuals *k*, $0\leq p_{c}\leq 1$ is the crossover probability and *a* is the minimum probability for individuals |k|:
  (18) $$a=1-p_{c}+p_{c}/N.$$
- Mutation operator:
  (19) $$P_{M}^{t}\left(i,j\right)=p_{m}^{d(i,j)}{\left(1-p_{m}\right)}^{N-d(i,j)}$$
  where $0\leq p_{m}\leq 1$ is the mutation probability, d(i,j) is the Hamming distance between *i* and *j* and *b* is the minimum probability:
  (20) $$b={\left(1-p_{m}\right)}^{N}.$$
- Selection operator: An MOEPT uses the strategy of retaining the elite options, and the best individual is retained for the next generation which does not participate in the competition. Assume that *m* individuals are selected based on the following equations:
  (21) $$P_{S}^{t}\left(P_{t},P_{t}^{j}\right)=\frac{\sigma_{n}\left(F\left(P_{t}^{j}\right)\right)}{\sum_{k=1}^{n_{max}}\left(F\left(P_{t}^{j}\right)\right)},j\in P_{t},n=1,2,\ldots .$$
  where $\sigma_{n}$ represents an increasing scale function. That aside, the probability of selecting the first individual in the next generation’s population is
  (22) $$P_{S}^{t^{*}}\left(P_{t},P_{t}^{j}\right)=\frac{|P_{t}|}{\left|B(P_{t})\right|},P_{t}\in P_{t}.$$
  where $|P_{t}|$ is the number of individuals $P_{t}$ in $P_{t}$ and $B(P_{t})$ is the cardinality of the optimal set of $P_{t}$.

In order to facilitate the convergence analysis, the changing process of the fitness value $F(P_{t}^{j})$ is regarded as a Markov chain. If the MOEPT obtains the best individual $P_{t}^{j_{best}}$ in generation *t*, we can denote this as $\left\{\hat{F}\left(P_{t}\right)\right\}=P_{t}^{j_{best}}$. Then, all the other populations in t+1 generations will also reach the best fitness value due to the elite strategy [34]. Therefore, the Markov chain $\left\{\hat{F}\left(P_{t}\right)\right\}$ constitutes the lower martingale. According to the properties of the lower martingale and the convergence theorem of the lower martingale [35], convergence analysis of the MOEPT is converted into the convergence of $\left\{\hat{F}\left(P_{t}\right)\right\}$. The following three theorems are given, in which Theorem 4 is for proving that $\left\{\hat{F}\left(P_{t}\right)\right\}$ satisfies the conditions of the martingale theorem, Theorem 5 proves the global convergence of the MOEPT and Theorem 6 constructs three conditions for the convergence of the lower martingale so that the optimal solution can almost be obtained everywhere.

**Theorem** **1.** *The process of describing the values of the fitness functions in the MOEPT is a non-bounded martingale:*

(23) $$E\left\{\hat{F}\left(P_{t+1}\right)/P_{t}\right\}\geq \hat{F}\left(P_{t}\right)$$

**Proof.**  Because the algorithm retains the maximum fitness value of the previous generation for the next generation and does not participate in the genetic operation, the best individual mode is not destroyed, so the maximum fitness value of the next generation’s population will not be less than the maximum fitness value of the previous generation:

(24) $$E\left\{\hat{F}\left(P_{t+1}\right)/P_{t}\right\}\geq \hat{F}\left(P_{t}\right)>0$$

   □

**Theorem** **2.** *The MOEPT converges to the global optimal solution based on the probability, which is mathematically expressed by the condition.*

**Proof.**  When the population $P_{t}$ is updated to generation *t*, the minimum or best fitness is recorded as $P_{t}^{{}^{\prime }j_{best}}$, and the global optimal solution is noted as $F^{*}$, we assume that the MOEPT can converge to a global optimal solution at generation *t* such that

(25) $$\left\{\hat{F}\left(P_{t}\right)\right\}=F^{*}$$

   □

Based on Theorem 4, we have

(26) $$E\left\{\hat{F}\left(P_{t+1}\right)/P_{t}\right\}=F^{*}$$

This is defined by the following conditional expectation:

$$\begin{array}{cc} \; & E\{\hat{F}\left(P_{t+1}\right)/P_{t}\}=\\ \; & \sum_{i,j\in P_{t}}P_{C}^{t}\left(i\times j,y\right)\sum_{v}P_{M}^{t}\left(y,v\right)\sum_{k}P_{S}^{t}\left(v,k\right)\hat{F}\left(k\right)\geq \\ \; & \sum_{i,j\in P_{t}}P_{C}^{t}\left(i\times j,i\right)\sum_{v}P_{M}^{t}\left(y,v\right)\sum_{k}P_{S}^{t}\left(v,k\right)\hat{F}\left(k\right)\geq \\ \; & a\sum_{v}P_{M}^{t}\left(y,v\right)\sum_{k}P_{S}^{t}\left(v,k\right)\hat{F}\left(k\right)\geq \\ \; & a\sum_{v}P_{M}^{t}\left(y,y\right)\sum_{k}P_{S}^{t}\left(v,k\right)\hat{F}\left(k\right)\geq \\ \; & ab^{m}\left\{\sum_{k\in B(P_{t})}\left[P_{S}^{t}\left(v,k\right)-P_{S}^{t*}\left(v,k\right)\right]\hat{F}\left(k\right)+\sum_{k\in B(P_{t})}P_{S}^{t*}\left(v,k\right)\hat{F}\left(k\right)\right\} \end{array}$$

When $k\notin B\left(P_{t}\right),P_{S}^{t*}\left(v,k\right)=0$, and when $k\in B\left(P_{t}\right),\hat{F}\left(k\right)=F^{*}$, $E\{\hat{F}\left(P_{t+1}\right)/P_{t}\}$ can be rewritten as

$$\begin{array}{cc} \; & E\{\hat{F}\left(P_{t+1}\right)/P_{t}\}\geq \\ \; & ab^{m}\sum_{k\in B(P_{t})}\left[P_{S}^{t}\left(v,k\right)\hat{F}\left(k\right)+F^{*}\right]\geq ab^{m}F^{*}. \end{array}$$

Therefore, we obtain

(27) $$ab^{m}F^{*}\leq F^{*}.$$

Because $F^{*}>0$, one obtains

(28) $$ab^{m}\leq 1.$$

Based on the above formula derivation, the MOEPT converges to the global optimal solution.

**Theorem** **3.** *When ∀n≥1, the following conditions are satisfied:*

- *$E\left[\hat{F}\left(P_{1}\right)\right]<\infty ,F^{*}<\infty$;*
- *$E\left[\hat{F}\left(P_{t}\right)/P_{t-1}\right]=\hat{F}\left(P_{t-1}\right)+c_{t-1}F^{*}$;*
- *$c_{t}\in \left[0,1\right],\lim_{t\to \infty }\sum_{k=0}^{t-1}c_{k}=1-\frac{\hat{F}\left(P_{1}\right)}{F^{*}}$.*

*Then, we have the random sequence $\hat{F}\left(P_{t}\right)\overset{a,s}{\to }F^{*}$*

Proof: By taking the mathematical expectation on both sides of condition (2), one has

$$\begin{array}{cc} \; & E\left[\hat{F}\left(P_{t}\right)\right]=E\left[\hat{F}\left(P_{t-1}\right)\right]+c_{t-1}F^{*}=\\ \; & E\left[\hat{F}\left(P_{t-2}\right)\right]+c_{t-1}F^{*}+c_{t-2}f^{*}=\ldots =\\ \; & E\left[\hat{F}\left(P_{1}\right)\right]+F^{*}\sum_{k=0}^{t-1}c_{k}. \end{array}$$

According to conditions (1) and (3), we have

$$\begin{array}{cc} \; & E\left[\hat{F}\left(P_{t}\right)\right]<E\left[\hat{F}\left(P_{1}\right)\right]+F^{*}<\infty \\ \; & Sup_{t}E\left[\hat{F}\left(P_{t}\right)\right]<sup_{t}E\left[\hat{F}\left(P_{1}\right)\right]+sup_{t}F^{*}<\infty \end{array}$$

Because $\hat{F}\left(P_{t}\right)$ is a non-bounded martingale, we have

(29) $$\hat{F}\left(P_{t}\right)\overset{a,s}{\to }\hat{F}\left(P_{\infty }\right)=\lim_{t\to \infty }\hat{F}\left(P_{t}\right)$$

$$\begin{array}{cc} \; & \lim_{t\to \infty }E\left[\hat{F}\left(P_{t}\right)\right]=\\ \; & \lim_{t\to \infty }E\left[\hat{F}\left(P_{1}\right)\right]+F^{*}\lim_{t\to \infty }\sum_{k=0}^{t-1}c_{k}=\\ \; & E\left[\hat{F}\left(P_{1}\right)\right]+F^{*}\left(1-\frac{\hat{F}\left(P_{1}\right)}{F^{*}}\right)=F^{*}, \end{array}$$

(30) $$\hat{F}\left(P_{t}\right)\overset{a,s}{\to }F^{*}.$$

## 5. Simulation Results

According to the first step of the MOEPT, we initially set the related parameters as follows: $t_{max}=50$, $n_{max}=1000$, $P_{s}=0.3$, $P_{c}=0.5$ and $P_{m}=0.1$.

### 5.1. Simple Examples

**Example** **3.** *Let us consider the frame $\Theta =\{\theta_{1},\theta_{2},\theta_{3},\theta_{4}\}$ and the corresponding BBA illustrated as follows:*

$$\begin{array}{cc} \; & m\left(\theta_{1}\right)=0.16,\;m\left(\theta_{2}\right)=0.14,\;m\left(\theta_{3}\right)=0.01,\;m\left(\theta_{4}\right)=0.02\\ \; & m\left(\theta_{1}\cup \theta_{2}\right)=0.20,\;m\left(\theta_{1}\cup \theta_{3}\right)=0.09,\;m\left(\theta_{1}\cup \theta_{4}\right)=0.04\\ \; & m\left(\theta_{2}\cup \theta_{3}\right)=0.04,\;m\left(\theta_{2}\cup \theta_{4}\right)=0.02,\;m\left(\theta_{3}\cup \theta_{4}\right)=0.01\\ \; & m\left(\theta_{1}\cup \theta_{2}\cup \theta_{3}\right)=0.10,\;m\left(\theta_{1}\cup \theta_{2}\cup \theta_{4}\right)=0.03\\ \; & m\left(\theta_{1}\cup \theta_{3}\cup \theta_{4}\right)=0.03,\;m\left(\theta_{2}\cup \theta_{3}\cup \theta_{4}\right)=0.03\\ \; & m(\Theta )=0.08 \end{array}$$

*Based on the respective classical PTs, the original BBAs are transformed into their corresponding probabilities as illustrated in Table 1. Their corresponding $C_{norm}$ values can be calculated using Equation (13), which is already listed in Table 1. Clearly, several interesting characteristics presented in Table 1 are worth mentioning: (1) $MOEPT_{d_{BI}^{E}+Sim_{seq}}$ had the minimum value from the perspective of the $C_{norm}$ criteria, which consider both aspects of $d_{BI}^{E}$ and $Sim_{seq}$ rather than concentrating on a single aspect, and (2) compared with other PTs, especially $MOEPT_{D_{J}+Sim_{seq}}$, (here, to show the property of $d_{BI}^{E}$, we replaced $d_{BI}^{E}$ with $D_{J}$ in the MOEPT to make comparisons) our method performed better than the mentioned methods. However, in practice, the suitability of various PTs depends on a number of factors, including the designer’s choices; that is, from the perspective of sequence similarity, $Sim_{seq}$ plays an important role in $C_{norm}$, but from the view of the whole distance, it involves transferring the principle role to $d_{BI}^{E}$ or $D_{J}$. How does one quantity this role? Here, we depend on the parameters $w_{1}$ and $w_{2}$ in $C_{norm}$ to distinguish our ideas from Han in [19], which initially set $w_{1}$ and $w_{2}$ as 0.5. Here, we discuss three different situations: (1). $w_{2}$ is set to 0.8 so as to pay more attention to the similarity of the sequence, (2) $w_{1}$ is set to 0.8 so as to focus more on the distance, and (3) considering both sequence similarity and distance, $w_{1}=w_{2}$ is set to 0.5, which is the same value used in [19], so that the similarity of both the sequence and distance are considered. This phenomenon, to some degree, reminds us of the importance of proper selections of weight in various applications when the MOEPT is applied. That aside, it is worth noting that $C_{norm}$ turned out to be $Sim_{seq}$ when $w_{1}=0$, and $C_{norm}$ turned out to be $d_{BI}^{E}$ when $w_{2}=0$.*

**Example** **4.** *Let us consider another situation in the frame $\Theta =\{\theta_{1},\theta_{2},\theta_{3},\theta_{4}\}$ and the corresponding BBAs illustrated as follows:*

$$\begin{array}{cc} \; & m\left(\theta_{1}\right)=0.16,\;m\left(\theta_{2}\right)=0.16,\;m\left(\theta_{3}\right)=0.16,\;m\left(\theta_{4}\right)=0.16\\ \; & m\left(\theta_{1}\cup \theta_{2}\right)=0.04,\;m\left(\theta_{1}\cup \theta_{3}\right)=0.04,\;m\left(\theta_{1}\cup \theta_{4}\right)=0.04\\ \; & m\left(\theta_{2}\cup \theta_{3}\right)=0.04,\;m\left(\theta_{2}\cup \theta_{4}\right)=0.04,\;m\left(\theta_{3}\cup \theta_{4}\right)=0.04\\ \; & m\left(\theta_{1}\cup \theta_{2}\cup \theta_{3}\right)=0.03,\;m\left(\theta_{1}\cup \theta_{2}\cup \theta_{4}\right)=0.03\\ \; & m\left(\theta_{1}\cup \theta_{3}\cup \theta_{4}\right)=0.03,\;m\left(\theta_{2}\cup \theta_{3}\cup \theta_{4}\right)=0.03 \end{array}$$

In actuality, Example 4 is the extension of the case studied by Han in [13], which assumes a special scenario where no difference exists between $m(\theta_{1})$, $m(\theta_{2})$, $m(\theta_{3})$ and $m(\theta_{4})$ and where the traditional PTs become invalid and give unreasonable results, which can be seen in Table 2. The property of the original BBA where no difference exists between $m(\theta_{1})$, $m(\theta_{2})$, $m(\theta_{3})$ and $m(\theta_{4})$ was almost lost when classical PTs were applied. When a “sequence” is not considered in an MOEPT, which is denoted as $MOEPT_{Distance}$, the feature of equal mass in the original BBAs was also missing, as with other classical PTs. Fortunately, when information of the “sequence” was added into the objective function, the MOEPT performed better in keeping the original information, as expected.

**Example** **5.**
$\Theta =\{\theta_{1},\theta_{2},\theta_{3},\theta_{4}\}$

To investigate the robustness of the MOEPT from a statistical point of view, in this example, we randomly generate BBAs and compare the MOEPT with classical PTs (BetP [6,26], CuzzP [12], DSmP [9], PrBP1 and PrBP2 [27]). The original BBAs for approximation are generated according to Algorithm 2 of [36].
**Algorithm 2:** Random generation of BBAs.1:Input: Frame of Discernment $\Theta =\{\theta_{1},\theta_{2},\theta_{3},\theta_{4}\}$  2:$N_{max}$:Maximum number of focal element  3:Output: BBA-m  4:Generate K(Θ), which is the power set of Θ  5:Generate a random permutation of K(Θ)→R(Θ)  6:Generate an integer between 1 and $N_{max}\to l$  7:For each First *k* elements of R(Θ) do  8:Generate a value within $\left[0,1\right]\to m_{i},i=1,\ldots ,l$  9:End  10:Normalize the vector $m=\left[m_{1},m_{2},\ldots ,m_{l}\right]\to m^{\prime }$  11:$m\left(\theta_{i}\right)=m_{i}^{\prime }$  

**Figure 2:**
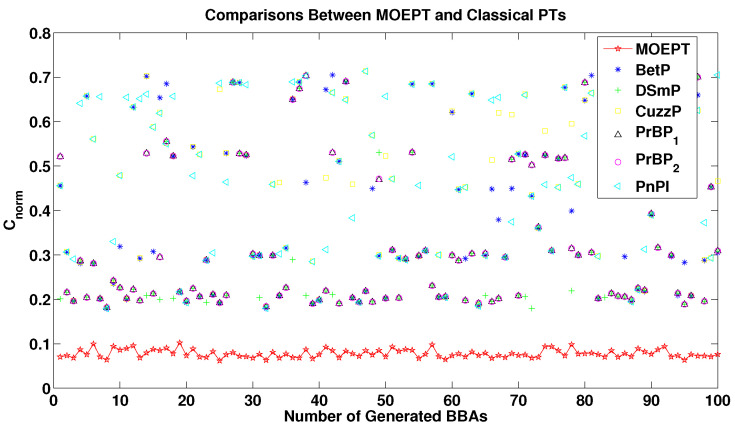
Comparisons between MOEPT and state-of-the-art PTs.

In our test, we set the cardinality of the FoD to 4 and fixed the number of focal elements to $l=N_{max}=15$. We randomly generated L=100 BBAs. Six PT methods were tested, and $C_{norm}$ was used to evaluate the quality of their corresponding results, shown in Figure 2. As we can see, and as was naturally expected, the MOEPT significantly outperformed the other methods based on the minimum $C_{norm}$ criterion, which is absolutely normal because the method was developed for this aim.

### 5.2. Example of Pattern Classification Using the MOEPT

**Figure 3:**
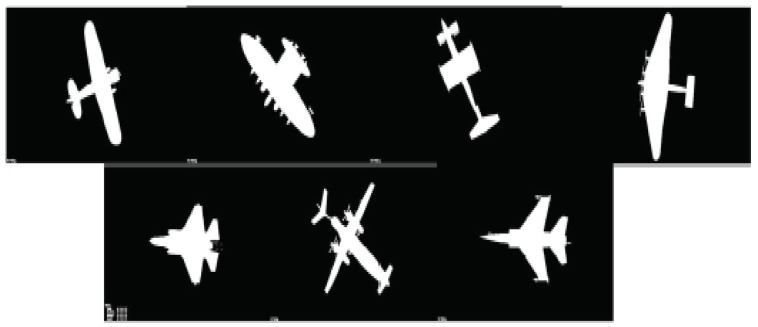
The binary images of seven kinds of airplanes.

In this example, we used the evaluation of decision making under the evidence theory framework to indirectly evaluate the MOEPT. We considered seven classes of aircraft, which are illustrated in Figure 3, and the classifier used in this example was the probabilistic neural network (PNN). For each test example, the output of the classifier was represented by a BBA. The corresponding BBA for each test sample was generated according to Li’s previous work [37].

1. First, the image was preprocessed with binarization, and then multiple features were extracted, such as Hu moments, the normalized moment of inertia, affine invariant moments, discrete outline parameters and singular values. Secondly, five BBAs could be assigned to the evidence sources for each PNN. (Specifically, the transfer functions in five PNNs were set to a Gaussian function, the weighting function was set to the Euclidean distance; the input function was netprod, and the output function was compet.) Third, all five of these BBAs were fused by PCR6 [7] to form a single BBA m(·).

2. For the two classes $t_{1}$ and $t_{2}$ ($t_{1},t_{2}\in 1,2,3,\ldots ,7,t_{1}\neq t_{2}$), with the top two values of m(i),i=1,2,3,…,7, the corresponding updated mass assignments were generated according to [38]:


(31) $$m^{\prime }\left(i\right)=m\left(i\right),\forall i=t_{1},t_{2}$$

The remaining mass was assigned to the total set Θ:

(32) $$m^{\prime }\left(\Theta \right)=1-m^{\prime }\left(t_{1}\right)-m^{\prime }\left(t_{2}\right).$$

For example, for a test sample target−1, we obtained the corresponding BBA from the PNNs, where m(1)=0.7,m(2)=0.05,m(3)=0.2,m(4)=0.01,m(5)=0,m(6)=0.02 and m(7)=0.02. The dominant class was class 1, and class 3 was in second place. The updated corresponding BBA was $m^{\prime }\left(1\right)=0.7,m^{\prime }\left(3\right)=0.2$ and $m^{\prime }\left(2,4,5,6,7\right)=0.1$.

There were 100 samples for each class, with a total of 700 samples. For each class, 50 samples were randomly selected for training PNNs, and the remaining samples were used for testing. For the MOEPT, the decision result would be class $t_{final}$ if

(33) $$t_{final}=argmax\left(MOEPT\right)$$

**Figure 4:**
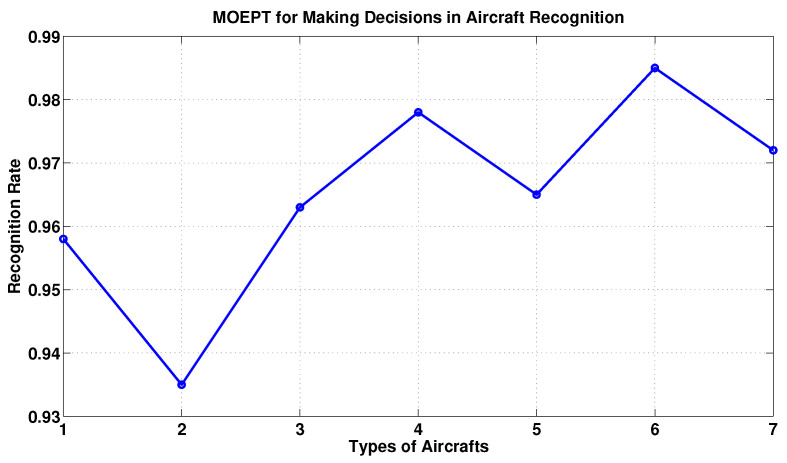
Recognition rate of MOEPT.

As we can see from Figure 4, the MOEPT performed well in this task of pattern classification.

### 5.3. Example of Target Type Tracking Using the MOEPT

To further discuss the practicality of the proposed MOEPT, a target type tracking (TTT) problem in the area of decision making was used, which is briefly described below [39].

#### 5.3.1. Target Type Tracking Problem (TTT)

1. Consider $\zeta =1,2,\ldots ,\zeta_{max}$ as the time index, and let there be *N* possible target types $Tar_{\zeta }\in \Theta =\left\{\theta_{1},\theta_{2},\ldots ,\theta_{N}\right\}$ in the surveillance area. For instance, in normal air target surveillance systems, the FoD could be $\Theta =\left\{Fighter,Cargo\right\}$; that is, $Tar_{1}=\theta_{1}≜Fighter$, and $Tar_{2}=\theta_{2}≜Cargo$. Similarly, the FoD in a ground target surveillance system could be $\Theta_{ground}=\left\{Tank,Truck,Car,Bus\right\}$. In this paper, we just considered the air target surveillance systems to prove the practicability of EPT.

2. At every time ζ, the true type of the target $Tar\left(\zeta \right)\in \Theta$ was immediately observed by an attribute-sensor (here, we assumed a possible target probability).

3. A defined classifier was applied to process the attribute measurement of the sensor, which provided the probability $Tar_{d}\left(\zeta \right)$ for the type of observed target at each instance ζ.

4. The sensor was, in general, not totally reliable and was characterized by an N×N confusion matrix:

(34) $$M=\left[M_{ij}=P\left(Tar_{d}=Tar_{j}|TrueType=Tar_{i}\right)\right]$$

where 0≤i≤N;0≤j≤N.

Here, we briefly summarize the main steps of the TTT using MOEPT:

1. Initialization: Determine the target type frame $\Theta =\left\{\theta_{1},\theta_{2},\ldots ,\theta_{N}\right\}$ and set the initial BBA $m^{initial}\left(\theta_{1}\cup \theta_{2}\cup \ldots \cup \theta_{N}\right)=1$, since there is no information about the first target type that will be observed;

2. Updating the BBA: An observed BBA $m_{obs}\left(.\right)$ on the types of unknown observed targets is defined from the current target type declaration and confusion matrix M;

3. Combination: We combine the current BBA $m_{obs}\left(\cdot \right)$ with the initial BBA $m^{initial}\left(\cdot \right)$ according to the PCR6 combination rule [7]: $m_{PCR6}\left(\cdot \right)=m_{obs}\left(\cdot \right)⊕m^{initial}\left(\cdot \right)$;

4. Approximation: Use MOEPT(·) to approximate $m_{PCR6}\left(\cdot \right)$ into a Bayesian BBA;

5. Decision making: Make a final decision about the type of the target at the current observation time based on the obtained Bayesian BBA;

6. Updating the BBA: Set $m^{initial}\left(\cdot \right)=m_{PCR6}\left(\cdot \right)$, and increase the time index ζ=ζ+1 before going back to step 2.

#### 5.3.2. Raw Dataset of TTT

We tested our MOEPT-based TTT on a very simple scenario for a 2D TTT problem, namely $\Theta =\left\{Fighter,Cargo\right\}$, for two types of classifiers. The matrix $M_{1}$ corresponds to the confusion matrix of the good classifier, and $M_{2}$ corresponds to the confusion matrix of the poor classifier:

(35) $$M_{1}=\left[\begin{array}{cc} 0.95 & 0.05\\ 0.05 & 0.95 \end{array}\right];M_{2}=\left[\begin{array}{cc} 0.75 & 0.25\\ 0.25 & 0.75 \end{array}\right]$$

**Figure 5:**
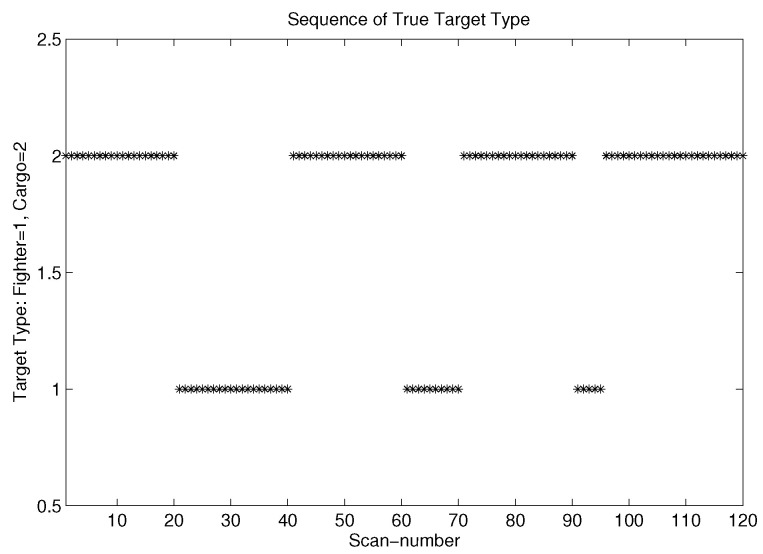
Raw sequence of true target type.

In our scenario, a true target type sequence over 120 scans was generated according to Figure 5. We can observe clearly from Figure 5 that Cargo (which is denoted as Type 2) appeared first in the sequence, and then the observation of the target type switched three times onto the Fighter type (Type 1) during different time durations (namely 20 s, 10 s and 5 s).

**A pathological case for TTT:** Our analysis showed that MOEPT can nevertheless be troublesome for tracking two target types, as proven in this particularly simple example (when $0\leq m(\theta_{1}\cup \theta_{2})\leq 0.1$). Let us consider the following BBA:

$$m_{target}\left(.\right)=\left[\theta_{1},\theta_{2},\theta_{1}\cup \theta_{2}\right]=\left[0,1,0\right]$$

According to the compatibility constraints in Equations (14)–(16), the population $P_{t}^{\prime }$ was obtained from $P_{t}$ through a selection procedure. Next, an individual ${P_{t}^{\prime }}_{j}$ in $P_{t}^{\prime }$, which is denoted as ${P_{t}^{\prime }}_{j}=\left[m^{\prime }\left(\theta_{1}\right),m^{\prime }\left(\theta_{2}\right)\right]$, was subjected to the initial constraint in Equations (1) and (36):

(36) $$\begin{array}{c} m^{\prime }\left(\theta_{1}\right)\geq \left(Bel\left(\theta_{1}\right)=m\left(\theta_{1}\right)=0\right)\\ m^{\prime }\left(\theta_{1}\right)\leq \left(Pl\left(\theta_{1}\right)=m\left(\theta_{1}\right)+m\left(\theta_{1}\cup \theta_{2}\right)=0+0=0\right);\\ m^{\prime }\left(\theta_{2}\right)\geq \left(Bel\left(\theta_{2}\right)=m\left(\theta_{2}\right)=1\right)\\ m^{\prime }\left(\theta_{2}\right)\leq \left(Pl\left(\theta_{2}\right)=m\left(\theta_{2}\right)+m\left(\theta_{2}\cup \theta_{1}\right)=1+0=1\right); \end{array}$$

From the above inequalities, one can see that only one probability measure, $P_{t}^{S}=\left[m\left(\theta_{1}\right),m\left(\theta_{2}\right)\right]=\left[0,1\right]$ (where the superscript index *S* means *single*), satisfied this constraint (the constraint was $m\left(\theta_{1}\right)\in \left[Bel\left(\theta_{1}\right),Pl\left(\theta_{1}\right)\right]=\left[0,0\right]$,$m\left(\theta_{2}\right)\in \left[Bel\left(\theta_{2}\right),Pl\left(\theta_{2}\right)\right]=\left[1,1\right]$). However because of the mechanism of MOEPT Equations (14)–(16), the $P_{t}^{j}$ in population $P_{t}$, which was randomly generated in the interval $\left[Bel\left(\theta_{i}\right),Pl\left(\theta_{i}\right)\right],i=1,2,\cdot ,N$, would be unable to generate enough candidates for evolutionary computation. (A sufficient number of candidate sets is a prerequisite for ensuring the global optimization performance of evolutionary algorithms.) That is why MOEPT becomes inefficient in this case, which occurs with a probability of $1/n_{\max}$, where $n_{\max}$ denotes the size of the population $P_{t}$. (In our simulation, we had $n_{\max}=1000$.) Unfortunately, in TTT decision-making problems, such a case cannot be avoided because it can really happen.

To circumvent this problem and make the MOEPT approach work in most circumstances, we needed modify the MOEPT method a bit to generate enough individuals for making the selection steps efficient when the bounds of the belief interval $\left[Bel,Pl\right]$ took their minimum and maximum values ($\left[0.9,0.05,0.05\right]$ and $\left[0.05,0.9,0.05\right]$, respectively). To achieve this, we proposed enlarging this particular interval through a parameter λ and maintaining the property of the original interval to some degree at the same time. More precisely, the modified belief interval, denoted as $\left[Bel^{\prime },Pl^{\prime }\right]$, was heuristically computed by a simple thresholding technique as follows.

First, we assume that the original BBA we consider here for the FoD ($\Theta =\{\theta_{1},\theta_{2}\}$) is $\left[\theta_{1},\theta_{2},\theta_{1}\cup \theta_{2}\right]=\left[a,b,c\right]$, with (a+b+c) = 1 and 0≤c≤0.1:

Step 1: Let $m^{\prime }\left(\theta_{1}\cup \theta_{2}\right)=c+\lambda$;
Step 2: If a>b, then
  (37) $$\begin{array}{c} m^{\prime }\left(\theta_{1}\right)=a-\lambda ;m^{\prime }\left(\theta_{2}\right)=b;m^{\prime }\left(\theta_{1}\cup \theta_{2}\right)=c+\lambda ; \end{array}$$
Step 3: If a≤b, then
  (38) $$\begin{array}{c} m^{\prime }\left(\theta_{1}\right)=a;m^{\prime }\left(\theta_{2}\right)=b-\lambda ;m^{\prime }\left(\theta_{1}\cup \theta_{2}\right)=c+\lambda ; \end{array}$$

Therefore, the values of $\left[Bel^{\prime }\left(\theta_{1}\right),Pl^{\prime }\left(\theta_{1}\right)\right]$ and $\left[Bel^{\prime }\left(\theta_{2}\right),Pl^{\prime }\left(\theta_{2}\right)\right]$ can be calculated based on Equations (37) and (38), which are presented as follows. When a>b, we have

(39) $$\begin{array}{c} \left\{\begin{array}{cc} Pl^{\prime }\left(\theta_{1}\right) & =m\left(\theta_{1}\right)+m^{\prime }\left(\theta_{1}\cup \theta_{2}\right)=a-\lambda +c+\lambda =a+c;\\ Bel^{\prime }\left(\theta_{1}\right) & =1-Pl^{\prime }\left({\bar{\theta }}_{1}\right)=1-\left(b+c+\lambda \right)=a-\lambda . \end{array}\right. \end{array}$$

(40) $$\begin{array}{c} \left\{\begin{array}{cc} Pl^{\prime }\left(\theta_{2}\right) & =m\left(\theta_{2}\right)+m^{\prime }\left(\theta_{1}\cup \theta_{2}\right)=b+c+\lambda =b+c+\lambda ;\\ Bel^{\prime }\left(\theta_{2}\right) & =1-Pl^{\prime }\left({\bar{\theta }}_{2}\right)\\ & =1-(a-\lambda +c+\lambda )=1-(a+c)=b. \end{array}\right. \end{array}$$

When a≤b, we have

(41) $$\begin{array}{c} \left\{\begin{array}{cc} Pl^{\prime }\left(\theta_{1}\right) & =m\left(\theta_{1}\right)+m^{\prime }\left(\theta_{1}\cup \theta_{2}\right)=a+c+\lambda ;\\ Bel^{\prime }\left(\theta_{1}\right) & =1-Pl^{\prime }\left({\bar{\theta }}_{1}\right)\\ & =1-(b-\lambda +c+\lambda )=1-(b+c)=a. \end{array}\right. \end{array}$$

(42) $$\begin{array}{c} \left\{\begin{array}{cc} Pl^{\prime }\left(\theta_{2}\right) & =m\left(\theta_{2}\right)+m^{\prime }\left(\theta_{1}\cup \theta_{2}\right)=b-\lambda +c+\lambda =b+c;\\ Bel^{\prime }\left(\theta_{2}\right) & =1-Pl^{\prime }\left({\bar{\theta }}_{2}\right)=1-\left(a+c+\lambda \right)=b-\lambda . \end{array}\right. \end{array}$$

**Explanation:** Through step 1, one computes the total singleton mass one has in the entire BBA, and the threshold value of 0.9 allows one to evaluate if the percentage of the singleton mass is big enough or not. Here, we not only consider the unique extreme case $m_{target}\left(\cdot \right)=\left[\theta_{1},\theta_{2},\theta_{1}\cup \theta_{2}\right]=\left[0,1,0\right]$ but also other possible cases, such as $m_{target}\left(\cdot \right)=\left[\theta_{1},\theta_{2},\theta_{1}\cup \theta_{2}\right]=\left[0.0001,0.9998,0.0001\right]$. Why do we consider the concept of this percentage? Actually, the higher the percentage of singleton mass, the smaller the interval for $P_{t}^{j}$; in other words, the higher value of $m\left(\theta_{1}\cup \theta_{2}\right)$, the bigger interval for $P_{t}^{j}$, which can be seen in Equation (36). Then, step 2 and step 3 give the method for calculating the updated upper bound of the belief interval $\left[Bel^{\prime },Pl^{\prime }\right]$, and Equations (39)–(42) prove that the parameter λ determines the range of the interval. Next, we give two examples to show how the above method works.

**Pathological case one for TTT** (revisited with a modified MOEPT):

$$m_{target}\left(.\right)=\left[\theta_{1},\theta_{2},\theta_{1}\cup \theta_{2}\right]=\left[0.0001,0.9998,0.0001\right].$$

**Figure 6:**
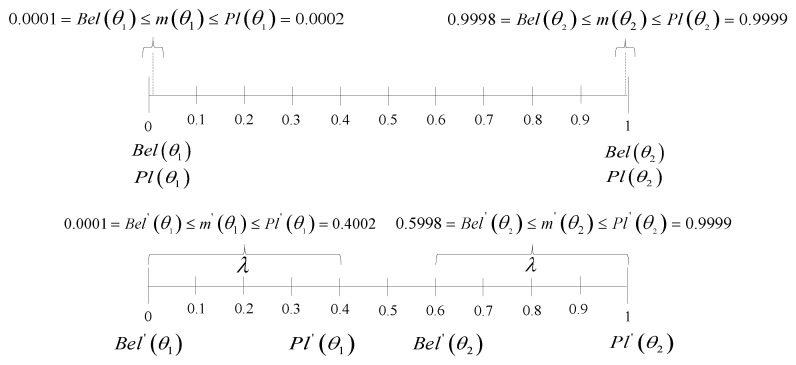
The principle of modified-constraint MOEPT (λ=0.4).

Here, the parameter λ is arbitrarily set to 0.4. (The value of the parameter λ can be chosen to be any value in $\left[0,1\right]$ by the designer for his or her own reasons to ensure the alternative interval effectively in the modified MOEPT.) Then, one computes in step 2 the modified plausibility bounds $Bel^{\prime }\left(\theta_{1}\right)=0.0001$, $Pl^{\prime }\left(\theta_{1}\right)=0.0001+0.0001+\lambda =0.4002$ and $Bel^{\prime }\left(\theta_{2}\right)=0.9998-0.4=0.5998$, $Pl^{\prime }\left(\theta_{2}\right)=0.9999$. Therefore, we obtain $\left[Bel^{\prime }\left(\theta_{1}\right),Pl^{\prime }\left(\theta_{1}\right)\right]=\left[0.0001,0.4002\right]$ and $\left[Bel^{\prime }\left(\theta_{2}\right),Pl^{\prime }\left(\theta_{2}\right)\right]=\left[0.5998,0.9999\right]$. The relationship between the original interval [Bel,Pl] and the updated interval $\left[Bel^{\prime },Pl^{\prime }\right]$ is illustrated in Figure 6.

Consequently, any Bayesian BBA $P_{t}^{j}=\left[m^{\prime }\left(\theta_{1}\right),m^{\prime }\left(\theta_{2}\right)\right]$ must be generated according the (modified) compatibility constraints:

$$\begin{array}{cc} m^{\prime }\left(\theta_{1}\right) & \in \left[Bel^{\prime }\left(\theta_{1}\right),Pl^{\prime }\left(\theta_{1}\right)\right]=\left[0.0001,0.4002\right]\\ m^{\prime }\left(\theta_{2}\right) & \in \left[Bel^{\prime }\left(\theta_{2}\right),Pl^{\prime }\left(\theta_{2}\right)\right]=\left[0.5998,0.9999\right] \end{array}$$

**Pathological case two for TTT** (revisited with a modified MOEPT):

$$m_{target}\left(.\right)=\left[\theta_{1},\theta_{2},\theta_{1}\cup \theta_{2}\right]=\left[0.45,0.48,0.07\right].$$

Here, the parameter λ is set to 0.2. Then, any Bayesian BBA $P_{t}^{j}=\left[m^{\prime }\left(\theta_{1}\right),m^{\prime }\left(\theta_{2}\right)\right]$ must be generated according the (modified) compatibility constraints:

$$\begin{array}{cc} m^{\prime }\left(\theta_{1}\right) & \in \left[Bel^{\prime }\left(\theta_{1}\right),Pl^{\prime }\left(\theta_{1}\right)\right]=\left[0.45,0.72\right]\\ m^{\prime }\left(\theta_{2}\right) & \in \left[Bel^{\prime }\left(\theta_{2}\right),Pl^{\prime }\left(\theta_{2}\right)\right]=\left[0.28,0.55\right] \end{array}$$

In order to evaluate the influence of parameter λ, we reexamined all the pathological cases based on the following procedure:

1. The value of parameter λ was set to five possible values: 0, 0.1, 0.2, 0.3, 0.4 and 0.5;
2. We randomly generated an initial population $P_{t}$ based on λ, which was also subject to the constraints in Equations (14)–(16).

**Figure 7:**
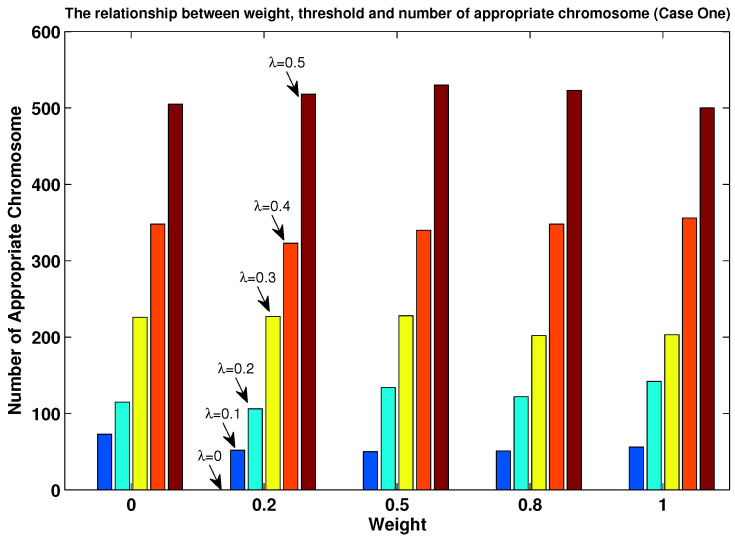
Impact of λ (x-axis) on individuals in $P_{t}$ (y-axis) for case one.

**Figure 8:**
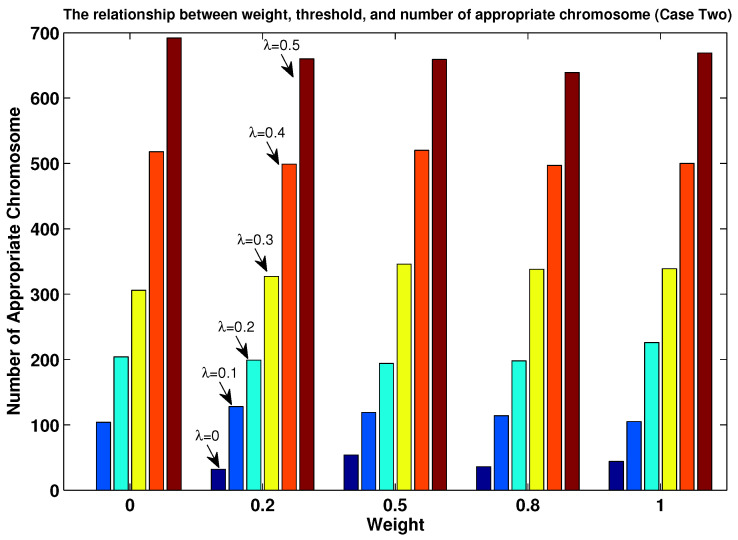
Impact of λ (x-axis) on individuals in $P_{t}$ (y-axis) for case two.

With this simulation, we can observe in Figure 7 and Figure 8 the impact of the λ value on the number of $P_{t}^{j}$ in $P_{t}$. When we set λ=0 (in which the original MOEPT was applied), there existed no suitable $P_{t}^{j}$ for case one, which demonstrates the necessity to circumvent the pathological case problem. Obviously, the number of $P_{t}^{j}$ increased with the increase in the λ value, which efficiently proves the advantage of using the modified MOEPT approach to make the selection step of the evolutionary algorithm more efficient. One point we need to clarify is that the intervals (i.e., $\left[Bel^{\prime }\left(\theta_{1}\right),Pl^{\prime }\left(\theta_{1}\right)\right]$, $\left[Bel^{\prime }\left(\theta_{2}\right),Pl^{\prime }\left(\theta_{2}\right)\right]$) induced from the parameter λ above aim at guaranteeing enough of a number of $P_{t}^{j}$ in $P_{t}$ in the implementation of the MOEPT. Another point we also need to mention is that the number of $P_{t}^{j}$ in $P_{t}$ was not influenced by the weight. (Here, the weight equals $w_{2}$ in Equation (13). And thus, $w_{1}=1-Weight$, which to some degree guarantees the implementation of the MOEPT.)

#### 5.3.3. Simulation Results of TTT Based on the Modified MOEPT

**Figure 9:**
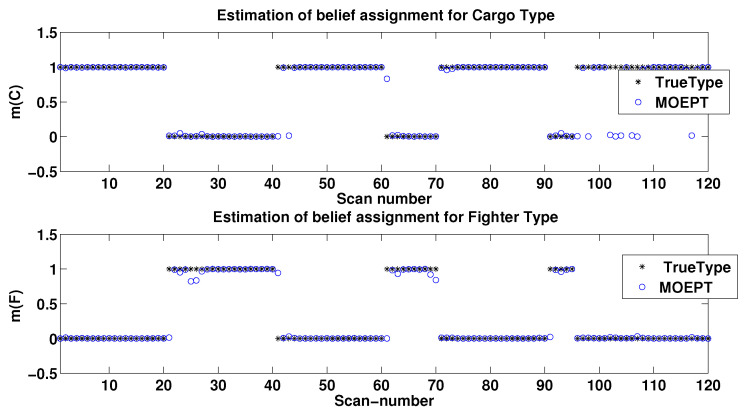
Results of MOEPT for Cargo and Fighter types using $M_{1}$.

**Figure 10:**
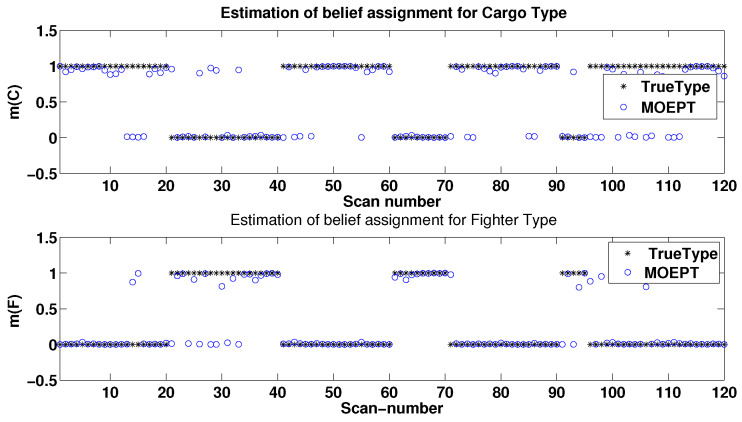
Results of MOEPT for Cargo and Fighter types using $M_{2}$.

Our simulation consisted of 100 Monte Carlo runs, and we show in the sequel the averaged performances of the MOEPT. Figure 9 and Figure 10 illustrate the Bayesian BBAs obtained by our new MOEPT method for solving the TTT problem using the PCR6 fusion rule. One can see that regardless of the good classifier $M_{1}$ (recognition rate: 90.83 %) or poor classifier $M_{2}$ (recognition rate: 80.83%) being used, the MOEPT was able to track properly the quick changes in target type.

## 6. Conclusions

A multi-objective evolutionary-based algorithm for probabilistic transformation (MOEPT) was proposed in this paper. It uses a genetic algorithm to obtain a Bayesian belief function and offer a comprehensive consideration concerning the closeness of distance between the orignal BBA and the Bayesian approximate one. In addition, a new aggregation measure was proposed in this paper to be combined into a more accurate “distance closeness” measure for MOEPT. More importantly, the convergence analysis of the MOEPT was given to prove the rationality of our proposed method. The effectiveness of the MOEPT was compared with respect to several probabilistic transformations proposed in the literature. Furthermore, the shortcomings of the original MOEPT version were clearly identified in two target type tracking problems, and they were solved thanks to modification of the belief interval constraints. As for future works, we would like to establish an adaptive scheme for the selection of weights in an MOEPT and make more comparisons between the performance of this MOEPT approach and other recently proposed evolutionary algorithms. We would also conduct more investigations to extend the MOEPT to DSmT using the DSm cardinal of elements. That aside, the current work in this paper is mainly for verifying the effectiveness of the algorithm through simulation examples from a theoretical perspective, and the feasibility of the proposed evolutionary-based PT will be verified through the practical and real problems in our future discussions.

## Figures and Tables

**Table 1:** Results of different PTs in Example 3 ($w_{1}$ = $w_{2}$ = 0.5).

	$\theta_{1}$	$\theta_{2}$	$\theta_{3}$	$\theta_{4}$	$C_{\mathrm{norm}}$
CuzzP	0.3860	0.3382	0.1607	0.1151	0.2800
BetP	0.3983	0.3433	0.1533	0.1050	0.2799
${\mathrm{DSmP}}_{0}$	0.5176	0.4051	0.0303	0.0470	0.1897
${\mathrm{DSmP}}_{0.001}$	0.5162	0.4043	0.0319	0.0477	0.1896
PrBP1	0.5419	0.3998	0.0243	0.0340	0.1918
PrBP2	0.5578	0.3842	0.0226	0.0353	0.1933
${\mathrm{MOEPT}}_{D_{J}+{\mathrm{Sim}}_{\mathrm{seq}}}$	0.3980	0.3322	0.1156	0.1541	**0.1849**
${\mathrm{MOEPT}}_{d_{\mathrm{BI}}^{E}+{\mathrm{Sim}}_{\mathrm{seq}}}$	0.3985	0.3983	0.0623	0.1409	**0.0733**

**Table 2:** Results of different PTs in Example 4.

	$\theta_{1}$	$\theta_{2}$	$\theta_{3}$	$\theta_{4}$	$C_{\mathrm{norm}}$
BetP	0.3983	0.3433	0.1533	0.1050	0.3974
$DSmP_{0}$	0.2500	0.2500	0.2500	0.2500	0.5458
PrBP1	0.5419	0.3998	0.0243	0.0340	0.6368
PrBP2	0.5578	0.3842	0.0226	0.0353	0.6412
$MOEPT_{Distance}$	0.2500	0.1597	0.3578	0.2325	0.3415
${\mathrm{MOEPT}}_{D_{J}+{\mathrm{Sim}}_{\mathrm{seq}}}$	0.2483	0.2485	0.2496	0.2536	0.1708
${\mathrm{MOEPT}}_{d_{\mathrm{BI}}^{E}+{\mathrm{Sim}}_{\mathrm{seq}}}$	0.2484	0.2488	0.2489	0.2539	**0.0450**

## Data Availability

Not applicable.
